# Elderly People’s Perceptions of Heat Stress and Adaptation to Heat: An Interview Study

**DOI:** 10.3390/ijerph19073775

**Published:** 2022-03-22

**Authors:** Anna Malmquist, Mattias Hjerpe, Erik Glaas, Hulda Karlsson-Larsson, Tina Lassi

**Affiliations:** 1 Department of Behavioural Sciences and Learning, Linköping University, SE-581 83 Linköping, Sweden; hulda.karlsson@liu.se (H.K.-L.); tinabranin@gmail.com (T.L.); 2 Department of Thematic Studies—Environmental Change, Center for Climate Science and Policy Research, Linköping University, SE-581 83 Linköping, Sweden; mattias.hjerpe@liu.se (M.H.); erik.glaas@liu.se (E.G.)

**Keywords:** heat, heatwave, elder, elderly care, perceptions, adaptation

## Abstract

Objectives: Heatwaves are having a disproportionate impact on the elderly population, as demonstrated by pronounced mortality and morbidity. The present study aimed to explore elders’ subjective experiences of heat impacts and adaptive strategies. Methods: Semi-structured interviews with 19 elderly Swedes were conducted, focusing on their experiences of the extremely hot summer of 2018. Results: Most informants suffered during the heatwave, although some found it pleasant. The readiness to implement adaptive measures was generally high among the healthiest, who were able to avoid excessive heat and adjust their daily routines. In contrast, those highly dependent on care from others had limited options for avoiding the heat, and little capacity to take up adaptive measures. Discussion: With heat becoming an increasing problem, it is important to adjust elderly care so that the most vulnerable elderly people can avoid excessive heat exposure.

## 1. Introduction

Despite Sweden’s temperate climate, heatwaves have been shown to significantly increase mortality (by 10–15%) [1], and in the summer of 2018, they probably caused over 600 excess deaths in the country [2]. In the last decade, the daily summer maximum temperature has increased by 1.7 °C, and as climate projections suggest more pronounced warming in the northern latitudes, heat events are expected to become more frequent [3].

The elderly, in particular those over 80 years of age, are among the most vulnerable when exposed to heat stress [2,4,5]. This is due to a range of factors. First, the aging body becomes more vulnerable to heat, and second, chronic diseases commonly associated with old age, and medications, increase this sensitivity [6]. Functional disability further decreases individuals’ readiness and options to implement measures to adapt to the heat [7]. For those who rely on external care to manage their personal hygiene and/or physical mobility, heat adaptation is highly dependent on access to sufficient care [8]. Further, social isolation and low socioeconomic status make elders’ heat adaptation even more difficult [8,9]. Measures to limit heat exposure, such as installing air conditioning, can prove too expensive. Psychologically, heat stress can cause worry, anxiety, and despair among vulnerable elders [8].

All in all, elderly people with health conditions, who are socially isolated and dependent on care from others, often experience significant stress during heatwaves, affecting their health and well-being. With this in mind, it is also important to acknowledge that the experience of heat is highly individual, and many elderly people do not perceive themselves as vulnerable to heat, in particular if they are socially active and healthy [10,11,12,13,14,15].

Several epidemiological studies have been conducted in Sweden, focusing on how heat affects the oldest among the elderly [1,2]. However, there is a lack of studies on how elderly Swedes perceive and adapt to extreme heat. Therefore, the present study aimed to explore elderly people’s subjective experiences of heat impacts and adaptive strategies. Specifically, semi-structured interviews with 19 elderly Swedes were conducted, focusing on their experiences of the extremely hot summer of 2018.

### 1.1. Heat Vulnerability among the Elderly

Elderly people are generally recognized as a group particularly vulnerable to heat. Epidemiological studies have established relationships between temperature and mortality and morbidity [1]. A range of individual factors, including old age and high physiological susceptibility due to pre-existing health conditions or use of medications, heat-protective behavior, and factors associated with socioeconomic status, such as living alone, housing conditions, and social contacts, are all associated with pronounced heat mortality and morbidity [14]. In addition to these epidemiological studies, there is a growing number of studies on subjective experiences of heat stress [10,12,14,16,17,18,19,20]. These studies find that elderly people often do not associate heat vulnerability with old age per se, but associate heat stress with social isolation, health impairments, reduced mobility, asset context and diversity, feelings of being helplessly exposed to heat, and housing conditions such as poor insulation, living on the top floor or in the attic, and limited access to green space and cooling devices. The studies generally find an uneven distribution of risk [12] and establish that socially active and healthy elderly people generally describe themselves as relatively unaffected by heat [10,12,14], whereas other individuals report severe impacts. Studies have also found that healthy elderly people often report a high adaptive capacity due to their freedom from the constraints of a working life, making it easier to change daily routines [10,14,16].

Studies of the subjective experience of heat have clearly established that heat can have impacts on health and aggravate social isolation [8], as most elderly people stay at home during extreme heat [12]. Social isolation can be viewed as the strongest driver of heat vulnerability [18], and it contributes to increased risk, as socially isolated elderly people, to a higher extent, feel unprepared to adapt to heat [12]. Regular visits by, e.g., children and neighbors have thus been shown to be very important during heat events, as they are able to check up on the health status of the elderly person [19]. For socially isolated individuals, municipal and community staff can be “a vital lifeline” [10,19].

Moreover, an individual’s independence and bodily integrity have been found to be important for understanding their subjective experiences of heat [10,12,19]. Those living in residential and nursing homes are often highly reliant on the staff to provide adaptation responses to heat stress and are therefore particularly vulnerable [21]. A reluctance to ask for help can further increase this vulnerability [12]. For example, individuals who need physical assistance to go to the bathroom may be hesitant of drinking a sufficient amount of water during a heatwave, in particular if they feel uncomfortable when asking for help or assume that they are causing troubles for those who provide care.

The fact that vulnerability to heat is disproportionally distributed between individuals in society calls attention to equity and justice issues in climate adaptation [22,23,24]. It is important to address how welfare, goods, and opportunities can be distributed. In the climate change context, this has mostly been discussed as an unjust and inequitable distribution of the impacts of and vulnerabilities to climate change.

### 1.2. Studying Elders’ Perspective on Heat in Sweden

Sweden has an aging population, with the number of inhabitants above 80 years of age expecting to increase by 50 per cent in the next decade [25]. Elderly care is a legislated right in Sweden, and it must meet the needs of the elderly individual [26]. Municipalities are obligated to assess the needs of the elderly person and to offer care that meets these needs, either in the care recipient’s own home or in a nursing home. In 2020, 11 per cent of individuals in Sweden over 65 years of age received home care, and 5 per cent lived in nursing homes [27]. In the age group of 80 years and older, the proportion receiving home care increased to 30 per cent, and 16 per cent lived in nursing homes. Among people living in nursing homes, many experience a lack of privacy, autonomy, and freedom to make decisions regarding their care [28]. Additionally, staff in elderly care feel they have insufficient time to provide optimal and well-adjusted care [26].

Previous research points at elderly people’s interest in maintaining control over their life conditions, including the “when and how” of the care they receive [28,29,30]. As heatwaves are expected to become more frequent and intense, it is important to consider elderly people’s need for sufficient care and how this should be adapted. For this purpose, it is important to understand how elderly people themselves experience heat stress, and what adaptation strategies they find useful or desirable.

Ill health among elders is often underestimated in research, due to the most vulnerable not being included among informants [31]. As heatwaves affect the most vulnerable among the elderly the most [6], their voices are of particular interest. Therefore, the majority of the informants in the present study were recruited at nursing homes.

## 2. Materials and Methods

### 2.1. Informants

A total of 19 individual semi-structured interviews were conducted with 19 elderly people: 12 women and 7 men. Eleven of the informants, aged 61–93 years, lived in nursing homes located centrally in a middle-sized Swedish city. The remaining eight informants, aged 73–92, lived in their own homes, and four of them received home care services. The informants’ homes were located in middle-sized cities and in the countryside in different parts of Sweden.

### 2.2. Procedure

In order to recruit informants from nursing homes, one member of the research team contacted the supervisor of each nursing home, who in turn informed the staff about the study aim and procedure. In the next step, staff asked residents if they were interested in participating in an interview and mediated written information about the study. When the residents were positive about participation, a time was scheduled for the interview. Most interviews were conducted in the informant’s apartment, while a few were conducted in common areas at the nursing home, but closed off so that other residents and staff would not interrupt.

In order to also reach elders who resided in their own home, another research team member visited home care centers and social venues for elderly people. Written information about the study was handed out and mediated by the staff to people who received home care services or visited the social venues. The contact details of those who agreed to participate were returned to the researcher, who scheduled and conducted the interview.

The interviews were conducted in 2019, one year after the heatwave. All interviews followed a semi-structured interview guide where the informants were asked about their experiences of the 2018 heatwave, how they perceived the heat, what they had done to manage the situation, and if this adaptation had been sufficient. All interviews were audio recorded.

### 2.3. Transcription and Analysis

The analysis followed a six-step thematic analysis as developed by Braun and Clarke [32]. First, all interviews were transcribed verbatim by the interviewers, with pseudonyms replacing names. Second, the entire data set was coded inductively. Third, codes were collected into a single document and sorted into broad candidate themes. Fourth, the candidate themes were reviewed and revised. Fifth, all final themes were named. Sixth, excerpts were selected form the interviews to illustrate the themes. The selected excerpts were analyzed in detail while the Results section was written. Besides the present article, an extensive report of the findings can be found in [33].

## 3. Results

In the following, the findings from the study are reported in three major themes, each in turn consisting of three subthemes. The first theme focuses on the informants’ experiences of the 2018 heatwave. The second and third themes focus on the adaptation measures employed to combat the heat and heat stress, with the second theme focusing on reactive adaptation measures that have been sufficient, and the third theme depicting the experience of insufficient adaptation resulting in major heat stress.

### 3.1. Perception of Heat and Heat Stress

In this section, we present how the informants experienced the heatwave of 2018. As shown below, a few of them perceived the heat as mostly pleasant, but most experienced heat-related fatigue and/or other forms of heat-related stress. As shown in the following, heat experiences are rather individual: at some temperatures, some suffer while others thrive.

#### 3.1.1. Heat Is Pleasant

Some of the informants, both at the nursing home and among those living in their own homes, described the heat as predominantly comfortable or pleasant, or as preferable to cooler temperatures, some even describing themselves as sun worshipers (e.g., Linnea, aged 89, residing in her own home with home care assistance). A few further described heat as pleasant because they otherwise tend to feel cold. Heat is usually associated with summer, which in turn is commonly considered positive. Gisela recalled the heat during the summer of 2018 as pleasant:

*I know that people complain about the heat a lot, yeah, well, they like wonder “How are you doing?”, since it’s sunny, very sunny here. But for some reason, that I really don’t know myself, I didn’t suffer much from the heat […] I have never felt very warm in that sense. No. Rather, if anything, the opposite: cold. So, evidently one could imagine that this summer must have been pleasant for me, as I generally feel cold.*

(Gisela, aged 88, nursing home)

While many of Gisela’s neighbors and the nursing home staff suffered during the heat event, she claimed to have been alright. Likewise, Ivan, aged 93 and living in a nursing home, reported that he usually felt cold and had been relatively unaffected by the heat. Consequently, for those who otherwise often feel cold, heat can be a welcome and pleasant change.

A few informants also described how health problems related to colder weather were reduced during the heat event. Iris stated:

*I dislike the cold and that is because I get a terrible itchiness and a bit of body-aches too … I don’t feel well in the cold at home. The worst for me is the itchiness. Because it drives me crazy, really. Medically. I have to rub myself with prescription [ointment].*

(Iris, aged 73, extra care housing)

Warmer and more humid weather was associated with reducing Iris’ problems with chronic pain and itchy skin, and, consequently, she mostly welcomed the heat.

#### 3.1.2. Heat Makes You Fatigued

While only a few informants described the heat in mainly positive terms, most had suffered during the excessive heat. Many experienced fatigue due to the heat. Some of those who lived in their own homes described themselves as having good general heath and an active life but explained how heat-related fatigue had forced them to change their habits. Mina described this:

*It [the summer of 2018] was very hot and I dislike heat. So, I found it very strenuous. I feel worse when it gets very hot. You become powerless.*

(Mina, aged 73, own home)

The lack of energy Mina experienced during the heatwave was difficult for her and sharply contrasted her otherwise active life. Others, in particular those living at the nursing home, who already needed much support and care due to illness, an ailment, or lethargy, experienced the heat as extremely exhausting on top of their previous health problems. Ella talked about fatigue:

*I become fatigued, that can happen. And that is, I would say, I believe it’s the heat. So that is clear. Still, I would say, when you become this old, you might be tired anyway, but clearly, to some extent it is the heat that makes you fatigued.*

(Ella, aged 90, nursing home)

Ella was ambivalent in her explanation of her lack of energy. On the one hand, she attributed her fatigue to the heat, but on the other hand, she also experienced fatigue related to her age.

#### 3.1.3. Heat Is Stressful

Those who suffered the most from the heat experienced it as highly stressful. This was most pronounced among the informants at the nursing homes. Besides fatigue, they experienced sleeping difficulties, skin problems, excessive sweating, thirst, difficulty breathing, and a feeling of being unclean while not being able to wash or shower themselves. Torgny was using a wheelchair and lived at a nursing home. He experienced major problems during the heat event and described how sweat stuck to his body and his clothes became wet from sweat.

*You become sweaty so you cannot sleep. It smears on the body, and sweaters and such things get wet. And you may only shower once a week. Oh, I get help to wash my upper body and a little bit on my butt that I cannot do myself. But still, it feels difficult in general. Do not feel comfortable when the temperature reaches 25 degrees [Celsius], at that point my difficulties start to emerge. And then they escalate the warmer it gets, and it feels strenuous.*

(Torgny, aged 75, nursing home)

The problem with the high indoor temperature in the nursing home caused major suffering for many of the informants, who were highly dependent on the care they received, care that was not sufficiently adjusted to counter the heat-related stress. Decreased personal hygiene was one of the most serious problems described by several informants. Several also talked about difficulty breathing, and this was experienced as highly stressful. Among them was Gun-Britt:

*It is when your body is getting very sweaty […] when you get sweaty the heat makes your body burn. It is because of my MS disease. It burns when I’m sweating, oh. When it’s, a shower doesn’t help. It gets hard to breathe, yeah. […] Chest gets strained, hard to breathe.*

(Gun-Britt, aged 61, nursing home)

Gun-Britt, living with multiple sclerosis, had difficulties verbalizing her experiences. Still, she explained that the excessive heat had been highly stressful for her, as she had a hard time breathing. Gun-Britt explained that she avoided taking showers, because she felt pain when the water flowed over her body. Unfortunately, she experienced the same burning pain when she was sweating.

### 3.2. Adapting Sufficiently during the Heat

As most informants experienced the heat as more or less unpleasant, they all took measures to adapt to the situation. Some of them, mainly those living in their own home, found sufficient ways to adapt to the heat and largely experienced the situation as manageable. These informants reported few experiences of health impairments and only small effects on their general well-being.

#### 3.2.1. Adjusting Daily Routines

Heat-related fatigue was primarily counteracted through changes in daily routines. This was a common behavioral adaptation response reported by informants who otherwise described an active life. Mina described this:

*You do not have the energy to go outdoors. I’ll go inside then and stay indoors when it gets really, really hot. But as the evening emerges, you can go outdoors.*

(Mina, aged 73, own home)

Mina stated that she usually spent time working in her garden or took long walks in the daytime. The lack of energy she experienced during the heat event had been a difficult experience for her, but she could adapt by doing the same activities later in the evening, when the temperature had fallen.

Among the informants living in their own homes, several reported a high ability to control the indoor temperature and keep it at a comfortable level. As they adjusted their daily routines and stayed indoors during the hot hours, they experienced few health impairments during the heat. It is worth noting that most of them resided in rural locations, in buildings that resisted heat well. Others had access to air-source heat pumps, fans, or air conditioning to reduce the indoor temperature. Egon, who had installed an air-source heat pump, which could be utilized both for cooling and heating purposes, described his indoor temperature as comfortable:

*Yes, it was [comfortable]. It was about 21–22 degrees indoors. Yes, evidently it costs a bit, but hello […] it’s worth it!*

(Egon, aged 80, own home)

The air-source heat pump helped Egon avoid exposure to the heat while indoors. He emphasized that running the air conditioning had been well-invested money during the heatwave, but due to costs for purchasing, installing, and running an air-source heat pump, he stressed that not everybody could afford it.

Another commonly reported behavioral adaptation response was seeking shade outdoors. Access to shadowed areas outside was, however, very varied, affecting the informants’ ability to be outdoors during the daytime. Egon, aged 80 and living in his own home, stated that having his own garden made it easier for him to be outside in the shade. The interview material suggests that access is important for being able to use shadowed outdoor areas; in the interviews, this was indicated by references to one’s own garden, balcony, or terrace rather than public parks and gardens.

#### 3.2.2. Accepting Approaches

Besides making adjustments to daily routines and relying on cooling technologies to manage the heat, some informants reported adaptation strategies that could be described in terms of acceptance. For those who were able to attain a comfortable indoor temperature, acceptance was about having to stay indoors to avoid the heat. Arne lived in a ground-floor apartment that he described as cool. He generally liked to go outdoors in the middle of the day, when the sun is at its highest. As he uses a wheelchair, Arne needed assistance from the home care staff to go outdoors. In the summer of 2018, he had to stay indoors more often:

*Well, I sat here by my window and saw that it was so sunny. I have got the thermometer there, so I could see that it was hot. […] But in here, it was cool, yes indeed.*

(Arne, aged 80, own home with home care assistance)

Despite having to stay inside more often, Arne expressed satisfaction in staying in his cool apartment and gazing through the window during the heatwave. He expressed no worries or discomfort about this, indicating that he had accepted that the weather cannot be controlled and therefore it was not worth spending so much energy thinking about it.

For those who reported uncomfortably high indoor temperatures, acceptance was about having to reduce their activities during the hot hours of the day, as Iris reflected:

*Iris: Well, I did not set the same standards [for household duties] for myself as I usually do.*

*Interviewer: You lowered the standards? Iris: I lowered the standards, definitely.*

*Interviewer: That’s not easily done for anyone.*

*Iris: No, not for anyone, but I can do that, surely. The main thing is that the sink and the bathroom and toilet are clean. Apart from them, it can accumulate.*

(Iris, aged 73, extra care housing)

Many, especially women who have been brought up to take good care of the home, set high standards for themselves regarding cleaning and keeping the household tidy. When it is hot and the person becomes lethargic, household chores can be difficult to keep up with. Iris expressed an accepting attitude that she had lowered the standards she set for herself.

The acceptance-oriented strategies were more commonly pronounced by the informants residing in their own homes who were relatively healthy and reported few health impairments during the heat. These people had more opportunities to adapt to the heat and thus could more easily adopt an accepting attitude.

#### 3.2.3. Social Contacts and Company

Contact with family and neighbors as well as municipal staff improved some of the informants’ ability to adapt during the heat. Some of the informants in this study mentioned that good company can help prevent loneliness and improve one’s ability to cope with the heat, as Egon recalled:

*Interviewer: Was there anybody around you who could help you cope with this heat?*

*Egon: Yes, possibly my children. They helped me reduce the pace. Brought the grandchildren. Then I could be indoors and take it easy […] And, above all, I had to go swimming. Especially when my daughter came with her children, then we had to go swimming outdoors. During the hottest period, I think we went every day.*

(Egon, aged 80, own home)

Egon mentioned two ways in which company improved his ability to manage during the heat. Firstly, being able to spend time with his children and grandchildren had made the time indoors, in his air-conditioned home, more enjoyable and meaningful. Secondly, accompanied by his daughter and grandchildren, he was able to go swimming in a nearby lake, something he said he would not have done on his own.

For those who do not have family or neighbors to socialize with, municipal staff are a source of social contacts. Karin, living in her own home with home care assistance, recalled how the staff reminded her to eat and drink enough:

*They [the home care staff] reminded me not to get sloppy and stop eating and all that. Because I actually wanted to [stop eating], because the food didn’t taste of anything. But I tried to eat a little, anyhow. And then this thing about drinking a lot, and that was good that they reminded me [to do that], otherwise I would probably have forgotten about that.*

(Karin, aged 82, extra care housing with home care assistance)

Because of her lack of appetite during the heat, Karin needed the kind reminders that she must eat and drink. Through her way of expressing herself, she gave the impression that she had experienced the staff’s care as benevolent but necessary. This is an example of how important it can be to have someone around vulnerable elderly people who care about them and encourage them to take care of their health, when they lack energy.

### 3.3. Inability to Sufficiently Adapt to the Heat

Several informants, especially among those at the nursing homes, explained that all adaptation measures they had utilized had been insufficient for them to manage the heat without suffering. Rather, they had experienced the heat as highly stressful, as described in the first theme.

#### 3.3.1. Limited Cooling Opportunities

The high indoor temperature at the nursing homes was stressful for many of the informants. Most of them described measures that they, their relatives, or the staff had taken to lower the indoor temperature or improve air circulation. Despite their efforts, these measures were insufficient. Some informants reported difficulties acquiring cooling equipment, as Gudrun recalled:

*Gudrun: It was hot, indeed, and I couldn’t get hold of a fan, because they were sold out. So I had to try to go outdoors and do a little of everything. I couldn’t use my balcony until late in the evening, because it was too hot. I have morning sun, so it gets really hot.*

*Interviewer: In what way was it difficult?*

*Gudrun: It was hard to go outdoors because I have some heart issues. I got so out of breath. And very fatigued.*

(Gudrun, aged 90, nursing home)

While Gudrun had to endure the heat without a fan, other informants did have fans but still experienced the indoor temperature as too high and uncontrollable. One of them is Martin, who stated:

*I had my fan on, I did, but it just makes the air go round and doesn’t help … I tried to stay in the shade and on the balcony … they [the staff] couldn’t do more. They cannot lower the heat either … They also thought that it was too hot.*

(Martin, aged 84, nursing home)

Torgny also noted that it was not possible to reduce the indoor temperature with a fan. According to him, the only thing that actually helped him was cross-ventilation:

*Cross ventilation is the only thing that actually works. But that is impossible at night. You don’t want to have your door open at night. And when everyone shuts their doors there is no cross ventilation […] I could not do anything else. I said afterwards that if I had known this, I would have bought a proper cooling device. But they were all sold out.*

(Torgny, aged 75, nursing home)

Torgny mentioned that the cooling devices were sold out during the heat event, but he also described another problem that particularly affected his ability to adapt to the heat at the nursing home, namely, that he must consider the needs and desires of the other residents. Opening doors or windows to enable cross-ventilation is rather easily achieved if you live in your own house, but at a nursing home, this requires coordination with others, who might not want it.

#### 3.3.2. Rumination and Prayer

When the informants experienced an insufficient ability to maintain the indoor temperature at a comfortable level, some reported that they were left with only rumination or prayer.

*Gun-Britt, who suffered badly during the hottest days, described her rumination:*

*Gun-Britt: Why, climate change. […]*

*Interviewer: Is that something you, like worry about, or think about a lot or so?*

*Gun-Britt: Doesn’t help. Thinking, but doesn’t help anyway.*

*Interviewer: No.*

*Gun-Britt: No, doesn’t help. I’m thinking but it doesn’t help.*

(Gun-Britt, aged 61, nursing home)

Gun-Britt said that the hot summer increased her thoughts and worry about climate change. She said that she had been reflecting a lot on climate change but also stated that her rumination did not help. In contrast to Gun-Britt, however, most informants did not worry about climate change. As they put it, they were approaching the end of their lives and—although the heatwave was in focus for the interview—they anticipated that they would not come to experience climate change. Moreover, Vendela said that she was praying that she would not experience another heatwave:

*It was just that I could not take it anymore, because it was very hot. My salvation was a cool apartment, that I locked myself into. Because it was indeed a very strenuous summer. And I have, as they say, been praying to God to not have this summer again. I just could not endure that.*

(Vendela, aged 90, nursing home)

Vendela was lucky to have access to a fan that she received as a birthday present. Therefore, her room was a bit cooler than the rest of the nursing home. She described the fan as her salvation and claimed that without it, she would not have survived the hot summer. When the next summer was approaching, she prayed to God that she would not have to experience something similar again. The quote above captures the pressed situation of the residents at the nursing homes. Isolating herself in her room can be assumed to have caused other negative consequences for Vendela, in that it reduces social interaction.

#### 3.3.3. Loneliness and Insufficient Care

As shown previously, some informants described having company as an important part of managing the heat. In addition, others said that loneliness exacerbated their situation. For many people, getting older means that they get lonelier, as partners and friends pass away. Loneliness was seen as a problem, especially for those who did not have children or other relatives who visited regularly. Jenny associated loneliness with her ability to adapt to the heat:

*Interviewer: Do you think heat affects you differently now that you are older compared to when you were young?*

*Jenny: Yes, of course it does. Like, I remember that we used to hang out at the beach, where we had our summer house. […] I went down there a lot, to swim and … but I cannot do that anymore. Like, you cannot drive to … Like, I cannot drive to the sea from here. And then when you are lonely, it is not that fun anymore, to drive to the sea.*

(Jenny, aged 76, own home)

Jenny, who used to have both a husband and children, said that she had lived a richer life when she was younger. She explained how she no longer drives to the sea on her own, even though she earlier had very much enjoyed this on hot summer days. It may be that she cannot find the motivation to do things that she previously associated with socializing now that she is on her own. This was claimed to the point that she said that she could not drive there, even though she usually used the car for other purposes. For Jenny, who experienced discomfort from the heatwave, the sea could have meant both coolness and joy, making life easier during the heatwave.

Another aspect that makes loneliness difficult is when you depend on external care to manage the heat. Some of the informants residing in nursing homes highlighted the staff’s lack of time to adapt caretaking during the heatwave. Torgny expressed his dissatisfaction with only getting one shower per week:

*Torgny: They do not have time for that.*

*Interviewer: No.*

*Torgny: There are not enough staff here at the nursing home.*

*Interviewer: No.*

*Torgny: All year around. The minimum limit, I think. Because in summertime, I would definitely want to shower at least twice a week.*

(Torgny, aged 75, nursing home)

As sweating more when it is hot is normal, more frequent showering should be a matter of course. Torgny believed that the reason why he was not allowed to shower more than once a week was a lack of staff and resources for elderly care. He noted that there was not enough staff to help all the people at the nursing home with more frequent showers. Torgny expressed his wish to shower at least twice a week, something that would have reduced the stress that the heat entailed.

## 4. Discussion

With heatwaves causing an increasing number of problems, measures to adjust care for the most vulnerable sections of the population are becoming an urgent problem [12,21]. In order to provide suitable adaptation measures, it is important to know how the elderly experience heat stress, as well as what adaptation measures they employ to manage the heat. The present study took the perspective of the elderly themselves, with the aim of providing their picture of experiencing and adapting to the excessive heat in Sweden during the summer of 2018. The majority of the informants in the present study lived in nursing homes and thus belonged to the most vulnerable among the Swedish elderly. Of the remaining informants, half received home care services. In total, most informants belonged to those elderly who are most vulnerable and sensitive when it comes to heat stress [6,15,21].

Most informants experienced the heat as highly unpleasant and stressful. Their testimonies of their suffering, in addition to previous research showing that heat stress causes serious physical and psychological health problems, including the risk of death [2,4,5,6], display the importance of addressing heat stress and implementing heat adaptation measures to protect the most vulnerable [8,12,24]. The variation among the informants, with some experiencing heat as mainly pleasant, thus showcases the huge individuality when it comes to experiencing heat, as also noted in previous research [10,11,12,13,14,15].

The adaptation measures described by the informants had different aims and focuses. Some measures were primarily directed at limiting heat exposure, through the usage of fans or ventilation, and by staying indoors. Such measures were available to and sufficient for some informants, but not for all. Informants living in their own homes had more opportunities to affect their indoor temperature and were generally better able to combat the heat. In contrast, several of the informants in the nursing homes experienced a hot indoor environment and had no, or insufficient, access to fans or other cooling systems. Previous research has shown that financial capacity affects elders’ ability to adopt heat-adapting measures (e.g., as not everyone can afford an air conditioning system) [8,9]. The present work adds another dimension to this, showing that the most vulnerable, living in nursing homes, had no or little opportunity to reduce their indoor temperature, merely because they no longer lived in a private home [21]. These informants found themselves in the uncomfortable situation of not being able to escape excessive heat exposure. This finding ties into previous research, showing how people in nursing homes suffer from limited freedom and autonomy to make decisions over their everyday life [28]. An unacceptable indoor temperature is just another area where lack of autonomy creates problems, in this case at the cause of a major health risk associated with the exposure to heat stress among the most vulnerable [2,4].

Another aspect of adapting to heat that was brought up by many informants concerned adjusting their activities to the weather conditions. Some informants described how they changed their daily routines: they had gone outdoors during early mornings or late evenings, rather than during the hottest hours. For elderly people who are able to go outdoors on their own, the rescheduling of a daily walk or a meeting with friends can be a good way to adapt to the conditions of heatwaves. As shown previously, those who are less mobile have more difficulties adapting to heat stress [7]. Thus, for those highly dependent on care, the service must be arranged to meet the need for adjusted routines. Previous research has stressed that elderly people want to keep control over their daily routines, a desire that is too often poorly met [28,29,30]. The importance of adjusting daily routines during a heatwave further underlines the need to individualize elderly care.

Several informants mentioned the importance of access to showers or baths during excessive heat. While access to good company was presented as important for those who regularly went swimming outdoors, for others, the lack of such company was a hindrance. Loneliness reduces quality of life among elderly people, and for those who are dependent on relatives for sufficient adaptation measures, loneliness lowers the capacity to adapt to heat stress [8,9,12,19]. According to the informants in the present study, access to company also affected their motivation to adopt heat-adapting measures (e.g., the motivation to take a refreshing bath, as well as the motivation to eat and drink in sufficient amounts).

Among the most vulnerable, access to showers is ultimately dependent on others. Informants at the nursing homes described the highly uncomfortable situation of being granted only one shower per week, even during the most excessive heat. This emphasizes the importance of adjusting care to the changed conditions during heatwaves [12] and pinpoints previously described problems of a lack of staff and time resources in Swedish elderly care [26].

Psychological coping was important for several informants who had found the heatwave manageable. They had been forced to adjust their daily routines or to stay indoors but still expressed an accepting attitude to these adjustments. In contrast, those who had not been able to take on sufficient measures to lower their heat exposure described being left only with rumination and prayer. Thus, while a change in daily routines had been found acceptable, not being able to escape the heat at all had been perceived as unbearable [8].

## 5. Limitations and Suggestions for Further Research

The present study reports findings from a small-scale interview study where nineteen elderly people in Sweden were interviewed about their experiences of the heatwave in the summer of 2018. This study has an explorative approach, focusing on areas that have previously not been explored. Therefore, the findings cannot be considered representative of the entirety of Sweden, as readiness to adapt to heat stress can vary among regions. To gain a more substantial picture of Swedish elders’ needs before future heatwaves, studies with broader and representative sampling are needed.

## 6. Conclusions

To conclude, the present article adds to research showing that many of the most vulnerable elderly people are exposed to unacceptable suffering during heatwaves, as they have little or no opportunity to escape excessive heat exposure. In contrast, among the healthier elderly people, several had found sufficient ways to adapt to the heat, including lowering heat exposure and changing daily routines. For the most vulnerable to benefit from similar strategies, elderly care must be offered in premises where the vulnerable residents are not exposed to excessive heat, and these premises must be able to be adapted to meet the individuals’ needs for adjustment of their care to the weather conditions.

## Data Availability

Interview data are not available for confidentiality reasons.
